# Anatomical Study of the Clavicles in a Chinese Population

**DOI:** 10.1155/2016/6219761

**Published:** 2016-03-21

**Authors:** Xu-sheng Qiu, Xiao-bo Wang, Yan Zhang, Yan-Cheng Zhu, Xia Guo, Yi-xin Chen

**Affiliations:** Department of Orthopaedics, Drum Tower Hospital, Medical School of Nanjing University, No. 321 Zhongshan Road, Nanjing 210008, China

## Abstract

*Background*. A reemergence of interest in clavicle anatomy was prompted because of the advocacy for operative treatment of midshaft clavicle fractures. Several anatomical studies of the clavicle have been performed in western population. However, there was no anatomical study of clavicle in Chinese population.* Patients and Methods*. 52 patients were included in the present study. Three-dimensional reconstructions of the clavicles were generated. The length of the clavicle, the widths and thicknesses of the clavicle, curvatures of the clavicle, the areas of the intramedullary canal, and sectional areas of the clavicle were measured. All the measurements were compared between genders and two sides.* Results*. The mean length of the clavicles was 144.2 ± 12.0 mm. Clavicles in males were longer, wider, and thicker than in females; also males have different curvatures in both planes compared with females. The men's intramedullary canals and sectional areas of the clavicle were larger than those of women. No significant difference between the sides was found for all the measurements.* Conclusion*. This study provided an anatomical data of the clavicle in a Chinese population. These clavicle dimensions can be applied to the modifications of the contemporary clavicle plate or a new development for the Chinese population.

## 1. Introduction

Fractures of the clavicle are common, accounting for 3% to 10% of all fractures and 35%–44% of fractures to the shoulder girdle [1–3]. The annual incidence of clavicular fractures is estimated to be between 29 and 64/100,000 people [1, 2, 4]. Fractures of the shaft account for 69–82% of all clavicular fractures, whereas fractures of the lateral and medial end of the clavicle account for 21–28% and 2-3%, respectively [5].

For the displaced shaft fractures, the traditional opinion has been that they rarely require operative stabilization. However, more recent studies, over the last ten years, have demonstrated the disadvantages of nonoperative treatment. The relatively high number of nonunions, weakness, persistent pain, and disappointing cosmetic results might lead to unsatisfactory results in conservatively treated patients, while the results of primary operative reduction and fixation have improved considerably [6–8]. Therefore there is a tendency towards surgical treatment for displaced shaft fractures.

Open reduction and internal plate fixation and intramedullary fixation are two of the most commonly used surgical techniques for treating displaced shaft fractures. Due to the complex and variable bony anatomy of the clavicle, an understanding of clavicular anatomy is paramount for optimal plate design and fracture fixation. Several anatomical studies of the clavicle have been performed in western population in recent years [9–12]. However, there was no anatomical study of clavicle in Chinese population in the literature. Since bone geometry differs among races [13–16], it is necessary to perform anatomical study of clavicle in a Chinese population for optimal plate design and fracture fixation.

## 2. Patients and Methods

52 patients (13 with pneumonia, 5 with lung bullae, 7 with lung tumor, and 27 with chest trauma) who underwent chest CT scan were included in the present study. None of the patients had previous fracture of clavicle. The average age was 49.8 ± 18.2 years. The population consisted of 52 (26 males, 26 females) right and 52 (26 males, 26 females) left clavicles. Informed consent was obtained from all study subjects. The study was authorized by the local ethical committee and was performed in accordance with the ethical standards of the 1964 Declaration of Helsinki.

### 2.1. CT Scan and Feature Measurement of the Clavicles

Patients were scanned using a light speed 16 pro spiral CT scanner (GE, Connecticut, USA) with 0.625 mm CT slices at 300 mA and 120 kV. The images were imported into the ADW4.2 work platform. Three-dimensional reconstructions of the clavicles were generated.

A reference coordinate system was established to measure the geometric features of the 3D reconstructed clavicle. The line that passed through central points of two articular surfaces was defined as the *x*-axis and was oriented outward; then the 3D reconstructed clavicle was rotated along the *x*-axis. The clavicle was projected onto the screen, and the projected area changed with the clavicle rotation. When the maximal projected area was reached, the plane parallel to the screen was defined as *xy*-plane, and the *y*-axis passed through the central point of sternoclavicular joint and was oriented anteriorly. The *z*-axis was perpendicular to the *y*- and *x*-axes, passed through the intersection of the *x*- and *y*-axes, and was oriented upward.

Several parameters were measured using the projection plane of the 3D clavicle into the *xy*-plane (axial plane or transversal plane). In this projection plane, one line connected two apices of the anterior and posterior edges of the lateral curvature; and the central point of this line was defined as the apex of the lateral curvature. Similarly, the apex of the medial curvature was defined.

**Figure 1:**
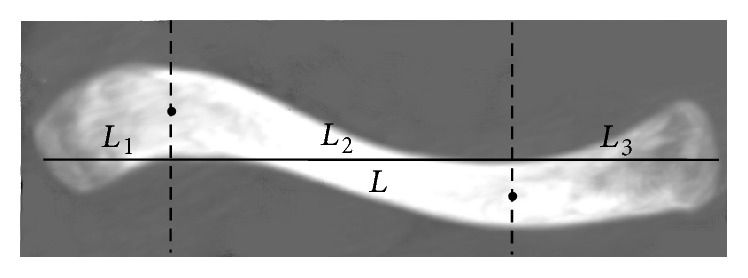
Measurements of the length (*L*), lateral segment (*L*_1_), middle segment (*L*_2_), and medial segment (*L*_3_) of the clavicle.

**Figure 2:**
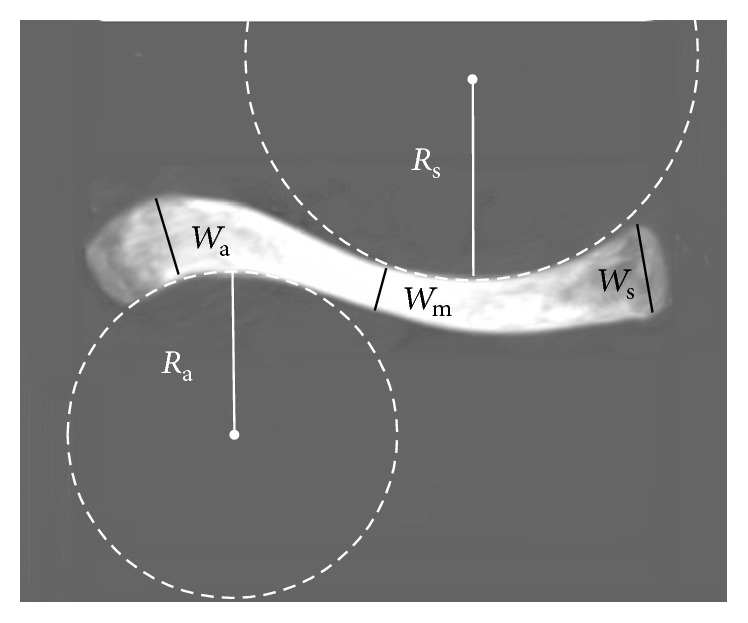
Measurements of the width and curvature of the clavicle in the axial plane. *W*_s_: the largest width at the sternal end; *W*_a_: the largest width at the acromial end; *W*_m_: the smallest width in the axial plane; *R*_s_: the radius of the circle fitted in the medial/sternal curvature; *R*_a_: the radius of the circle fitted in the lateral/acromial curvature.

**Figure 3:**
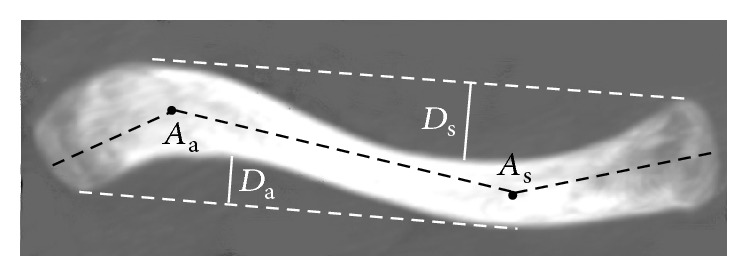
Measurements of the intersegmental angles and depth of the curvature in the axial plane. *A*_s_: the angle between the medial and middle segments; *A*_a_: the angle between the lateral and middle segments; *D*_s_: the depth of the medial/sternal curvature; *D*_a_: the depth of the lateral/acromial curvature.

**Figure 4:**
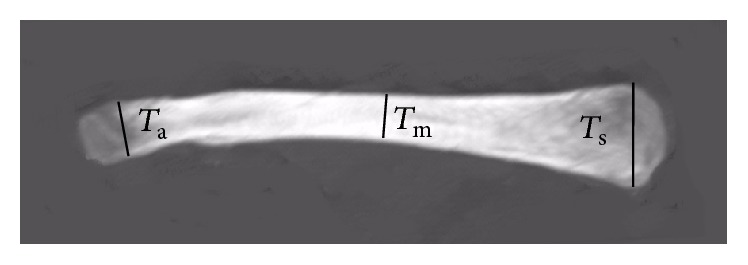
Measurements of the thickness of the clavicle in the coronal plane. *T*_s_: the largest thickness at the sternal end; *T*_a_: the largest thickness at the acromial end; *T*_m_: the smallest thickness in the coronal plane.

**Figure 5:**
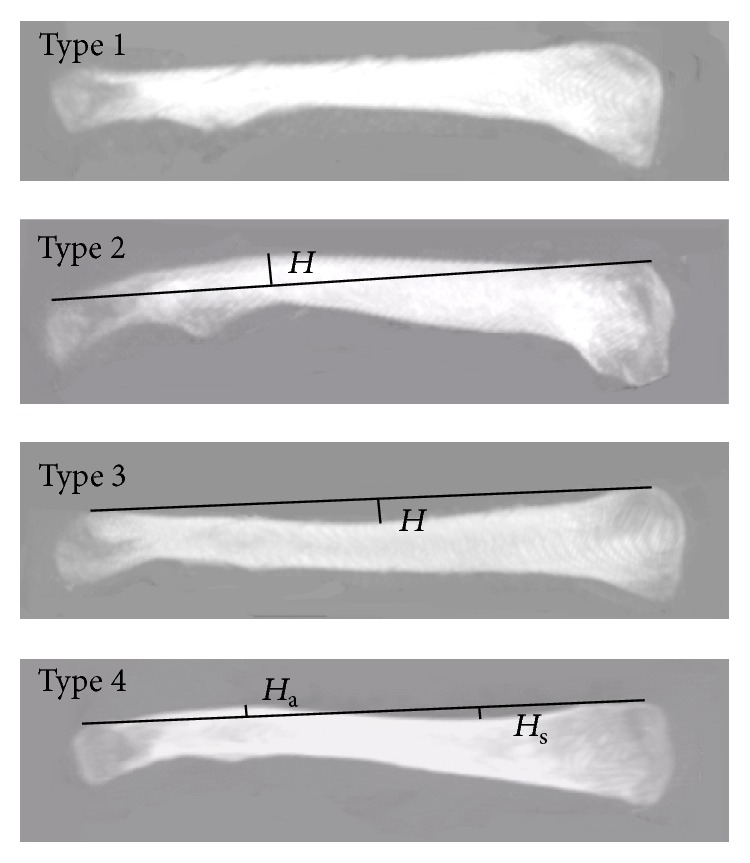
Measurements of curvature in the coronal plane. *H*: the height of the superior curvature; *H*_s_: the height of the superior curvature near the sternal end in type 4 clavicle; *H*_a_: the height of the superior curvature near the acromial end in type 4 clavicle.

**Figure 6:**
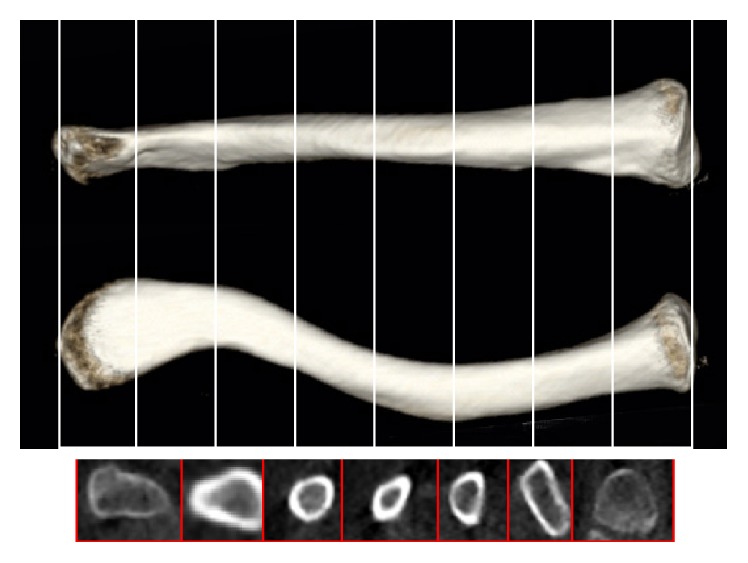
Measurements of the areas of the intramedullary canal (S) and sectional areas of the clavicle (OS).

(1) Length of the clavicle (*L*): the distance between the central points of two articular surfaces was measured and was defined as *L*. Each clavicle was divided into lateral segment (*L*_1_), middle segment (*L*_2_), and medial segment (*L*_3_) based on the medial and lateral apices of curvature (Figure 1).
(2) Width of the clavicle in the axial plane: one line is perpendicular to the anterior surface of the clavicle and passes through one point of the anterior surface and another point of the posterior surface. The distance between these two points was defined as the width of the clavicle in the axial plane. The largest width at the sternal end (*W*_s_) and at the acromial end (*W*_a_) was evaluated. The smallest width of the clavicle was also measured (*W*_m_). The location of these widths was measured and defined as a percentage of the length of the clavicle starting at the sternal end (Figure 2).
(3) Curvature of the clavicle in the axial plane:
  (a) Radius of curvature: the medial and lateral curvatures were fitted with circles most clearly suggesting an arc of curvature and their radii were measured (*R*_s_ for sternal/medial curvature and *R*_a_ for acromial/lateral curvature) (Figure 2).
  (b) Intersegmental angles: medial apex, lateral apex, and the central points of the sternoclavicular end and acromioclavicular end were connected with three lines; and these lines created intersegmental angles. The angle between the medial and middle segments was defined as *A*_s_. The angle between the lateral and middle segments was defined as *A*_a_ (Figure 3).
  (c) Depth of the curvature: one line connects the most posterior point of the medial end and the apex of the posterior edge of the lateral curvature. The perpendicular distance from the apex of the posterior edge of the medial curvature to this line was defined as the depth of the medial/sternal curvature (*D*_s_). Similarly, one line connects the most anterior point of the lateral end and the apex of the anterior edge of the medial curvature. The perpendicular distance from the apex of the anterior edge of the lateral curvature to this line was defined as the depth of the lateral/acromial curvature (*D*_a_) (Figure 3).
 Several parameters were also measured using the projection plane of the 3D clavicle into the *xz*-plane (coronal plane).
(4) Thickness of the clavicle in the coronal plane: one line is perpendicular to the superior surface of the clavicle and passes through one point of the superior surface and another point of the inferior surface. The distance between these two points was defined as the thickness of the clavicle in the coronal plane. The largest thickness at the sternal end (*T*_s_) and at the acromial end (*T*_a_) was evaluated. The smallest thickness of the clavicle was also measured (*T*_m_). The location of these widths was measured and defined as a percentage of the length of the clavicle starting at the sternal end (Figure 4).
(5) Curvature of the clavicle in the coronal plane: according to the superior curvature of the clavicle in the coronal plane, the clavicles were divided into four types: type 1, horizontal; type 2, convex, always on the acromial end; type 3, concave, always on the sternal end; type 4, S-shaped. One line connects two end points of the superior curvature. The perpendicular distance from the apex of the superior curvature to this line was defined as the height of the superior curvature (*H*). Positive was defined as the apex was above this line, and negative was defined as the apex was below this line. For type 4 clavicle, there were two apexes, one near the acromial end (*H*_a_) and the other near the sternal end (*H*_s_) (Figure 5).
(6) Along the *x*-axis, the clavicle was divided into 8 equal segments.* The areas of the intramedullary canal (S) and sectional areas of the clavicle (OS)* were measured in seven cross sections (Figure 6).

### 2.2. Interobserver and Intraobserver Measurement Variability

To test the interobserver and intraobserver variability of measurements, the parameters of 10 patients were measured by two observers (Wang XB, QIU XS) on two different occasions.

### 2.3. Statistical Analysis

The statistical analysis was performed by SPSS 13.0 for Windows. Pearson's rank correlation coefficients were used to assess measurement variability. Two-sample *t*-test was used to establish whether the differences of the mean values between genders were statistically significant. Paired *t*-test was used to establish whether the differences of the mean values between sides and segments were statistically significant. Statistical analysis of the distribution of the curvature types between genders was performed by *χ* ^2^ test. Statistical significance was set at *P* < 0.05.

## 3. Results

### 3.1. Length

The mean length of the clavicles was 144.2 ± 12.0 mm (121.7–173.2) for the general population. The mean length of the male clavicles was significantly longer (17.3 mm) than that of the female clavicles (*P* < 0.05). The mean length of the left clavicles was longer (1.4 mm) than the length of the right clavicle. However, the difference was not statistically significant (*P* > 0.05) (Table 1).

The mean lengths of *L*_1_, *L*_2_, and *L*_3_ were 39.7 ± 8.4 mm, 61.5 ± 7.1 mm, and 43.5 ± 8.7 mm, respectively. The mean length of *L*_2_ was significantly longer than *L*_1_ and *L*_3_ (*P* > 0.05), and there were no significant differences between *L*_1_ and *L*_3_ (*P* < 0.05). All three segments were significantly smaller in females than in males (*P* < 0.05). Again, there were no significant differences between left side and right side for all three segments (*P* > 0.05) (Table 1).

### 3.2. Width and Thickness

In the axial plane, the largest mean width at the sternal end was 22.1 ± 3.6 mm and was located at 4.3 ± 0.9% of length, while the largest mean width at the acromial end was 22.7 ± 4.1 mm at 94.7 ± 1.2%. The largest mean width was significantly smaller in female than in male clavicles at both ends (*P* < 0.05). The smallest mean width was 11.4 ± 2.1 mm in the axial plane and was located at 51.9 ± 4.6% of length. The smallest mean width was significantly thinner in females than in males (*P* < 0.05). No significant difference between the sides was found when measuring these three widths in the axial plane (Table 1).

In the coronal plane, the largest mean thickness at the sternal end was 20.8 ± 6.0 mm and was located at 4.9 ± 1.2% of length, while the largest mean thickness at the acromial end was 14.1 ± 3.8 mm at 95.8 ± 1.3%. The largest mean thickness was significantly smaller in female than in male clavicles at acromial end (*P* < 0.05), but not at sternal end. The smallest mean thickness was 10.5 ± 1.9 mm in the coronal plane and was located at 57.9 ± 5.3% of length. The smallest mean thickness was significantly thinner in females than in males (*P* < 0.05). No significant difference between the sides was found when measuring these three thicknesses in the coronal plane (Table 1).

### 3.3. Curvature

In the axial plane, the clavicle showed an S-shaped appearance. The circle fitted within the medial curvature had a mean radius of 63.2 ± 12.1 mm, while the radius of the circle fitted in the lateral curvature was 29.4 ± 8.2 mm. The difference in means was found to be statistically significant (*P* < 0.05). The medial intersegmental angle had a mean of 148.1° ± 5.4°, while the lateral intersegmental angle had a mean of 146.6° ± 8.7°. The mean medial depth of the curvature was located at 33.3 ± 2.5% and measured 17.4 ± 4.0 mm; the mean lateral depth of the curvature was located at 77.4 ± 2.4% and measured 12.7 ± 3.3 mm. For the medial curvature, the mean radius and depth of the curvature were significantly smaller in females than in males, while the mean medial intersegmental angle was significantly larger in females than in males. For the lateral curvature, the mean depth of the curvature were significantly smaller in females than in males, while there were no significant differences for the mean radius and intersegmental angle between males and females. There were no significant differences between left side and right side for all these curvature measurements (*P* > 0.05) (Table 1).

In the coronal plane, there were 3.8% (4/104) type 1 (horizontal), 32.7% (34/104) type 2 (convex), 13.5% (14/104) type 3 (concave), and 50.0% (52/104) type 4 (S-shaped) clavicles according to the superior curvature. The distribution of the curvature types was significantly different between males and females. For type 2, a convex curvature was present and the largest depth of the curvature was located at 63.4 ± 6.1% and measured as 4.5 ± 1.7 mm. For type 3, a concave curvature was present and the largest depth of the curvature was located at 37.1 ± 7.5% and measured as −5.7 ± 1.7 mm. For type 4, a concave curvature was present and the largest depth of the curvature was located at 25.0 ± 5.2% and measured as −2.7 ± 1.0 mm, and second convex curvature was present and the largest depth of the curvature was located at 70.7 ± 5.1% and measured as 3.0 ± 1.4 mm. The mean depth of the curvature was not significantly different between genders except the largest depth of the concave curvature for type 4. No significant difference was found between the two sides concerning the curvature in the coronal plane (Table 1).

### 3.4. Intramedullary Canal and Sectional Areas of the Clavicle

The intramedullary canal decreased gradually from two ends toward the center where it became the smallest. The men's medullary canals were larger than that of women. No significant difference was found between the two sides concerning the intramedullary canal. Similarly, the sectional areas of the clavicle decreased gradually from two ends toward the center. The men's sectional areas were larger than that of women. No significant difference was found between the two sides concerning the sectional areas (Table 2).

The Pearson correlation coefficients of interobserver repeat measurements ranged from 0.96 to 0.99, and the Pearson correlation coefficients of intraobserver repeat measurements ranged from 0.94 to 0.99. All correlations were statistically significant, and the level of measurement reliability was excellent.

## 4. Discussion

Midshaft clavicle fractures have traditionally been managed conservatively, but there is a reemergence of interest in operative fixation due to a number of reasons recent years [6–8]. Open reduction and internal plate fixation and intramedullary fixation are two commonly used surgical techniques for treating displaced shaft fractures [5, 17]. Plate fixation options include low contact dynamic compression plates, reconstruction plates, and precontoured locking plates. Precontoured locking plates have recently been introduced, matching the shape of the clavicle [5]. Some scholars have advocated the use of anatomical precontoured plates for the decrease in soft tissue irritation and increase in patient's satisfaction [18, 19]. VanBeek et al. [19] have compared the outcome of precontoured and noncontoured superior plating for displaced midshaft clavicle and showed a lower rate of plate prominence and less reoperation rate for hardware removal in the precontoured group.

To define the anatomical shape of the plate or to fix the fractured clavicle with intramedullary nails, a comprehensive understanding of the bony morphology is crucial. However, current morphologic studies focused on the western population [9–12]. There was no literature regarding the detailed anatomical data of the clavicle in a Chinese population. The current study provides the detailed anatomical data of the clavicle in a Chinese population and may be beneficial for better bone-plate fitness when designing the implant and providing useful data for intramedullary fixation in Chinese and Asian population.

The average clavicle length in our population was 144.2 mm, which is consistent with previous findings in western population (Table 3). This result implied that the shoulder width may be comparable between the Chinese population and the western population.

The lungs and subclavical artery are in close proximity to the clavicle. Knowing the width and thickness of the clavicle can be helpful to evaluate the screw length when anterior or superior plate and screw fixation are used. We measured the largest and smallest width and thickness of the clavicle in the axial and coronal planes. The results showed that the largest width and thickness located at the sternal end and acromial end, while the smallest width and thickness located at the clavicle shaft. Compared with Bernat et al.'s study [9], we find that Caucasian peoples have larger sternal thickness than Chinese peoples, while they have comparable or even smaller thickness at the acromial end and clavicle shaft in the coronal plane (Table 3). However, the widths in the coronal plane do not have this trend [9, 10].

In the axial plane, the clavicle showed an S-shaped configuration. Compared with western population (Table 3), we find that the radii of both (medial and lateral) curvatures of Chinese people were smaller than that of western people [10, 12], while the depth of the curvatures of Chinese people was larger than that of western people [10]. The findings can be important in the design of new anatomical clavicular plates.

In the literatures [9, 20], the bow in the coronal plane was mentioned; however, the inferior curvature or the central line curvature was measured. In the present study, the superior curvature was measured and it was divided into four types. To our knowledge, it is the first time to divide the clavicles into four types according to the superior curvature in the coronal plane. We thought the superior curvature was useful for the design of precontoured plate as most of the plates were placed superiorly. According to the results, four sets of plates may be required to improve bone-implant fitness. Furthermore, if four sets of plates are not available, it is also helpful for the surgeons to contour the plate to keep this clavicular anatomy in mind.

In conclusion, the results of this study provided an anatomical data of the clavicle in a Chinese population. These clavicle dimensions can be applied to the modifications of the contemporary clavicle plate or a newly development for the Chinese population. The statistics analysis also revealed significant differences in dimensions between male and female clavicle, indicating that distinguished designs for the genders may be required to improve bone-implant fitness.

## Figures and Tables

**Table 1:** The length, width, thickness, and curvature of the clavicle in Chinese population.

Measurements	Total	Male	Female	*P*	Left	Right	*P*
Length							
*L*	144.2 ± 12.0	152.9 ± 9.3	135.6 ± 7.2	*P* < 0.05	144.9 ± 11.9	143.5 ± 12.2	*P* > 0.05
*L*_1_	39.7 ± 8.4	42.2 ± 8.4	37.2 ± 7.7	*P* < 0.05	40.0 ± 8.8	39.3 ± 8.1	*P* > 0.05
*L*_2_	61.5 ± 7.1	64.5 ± 7.3	58.5 ± 5.3	*P* < 0.05	60.8 ± 6.8	62.2 ± 7.7	*P* > 0.05
*L*_3_	43.5 ± 8.7	46.8 ± 8.9	40.2 ± 7.2	*P* < 0.05	44.2 ± 9.5	42.7 ± 7.8	*P* > 0.05
Width in the axial plane							
*W*_s_	22.1 ± 3.6	24.1 ± 3.2	20.2 ± 2.8	*P* < 0.05	21.9 ± 3.9	22.3 ± 3.3	*P* > 0.05
*W*_m_	11.4 ± 2.1	12.7 ± 1.7	10.2 ± 1.3	*P* < 0.05	11.4 ± 1.9	11.5 ± 1.9	*P* > 0.05
*W*_a_	22.7 ± 4.1	25.1 ± 3.6	20.3 ± 2.9	*P* < 0.05	22.6 ± 4.2	22.8 ± 4.0	*P* > 0.05
Thickness in the coronal plane							
*T*_s_	20.8 ± 6.0	21.1 ± 7.5	20.6 ± 4.1	*P* > 0.05	21.1 ± 6.2	20.5 ± 5.8	*P* > 0.05
*T*_m_	10.5 ± 1.9	11.7 ± 1.9	9.3 ± 1.0	*P* < 0.05	10.5 ± 1.6	10.5 ± 2.2	*P* > 0.05
*T*_a_	14.1 ± 3.8	16.3 ± 3.8	11.9 ± 2.2	*P* < 0.05	14.0 ± 3.9	14.2 ± 3.7	*P* > 0.05
Curvature in the axial plane							
*R*_s_	63.2 ± 12.1	66.4 ± 12.8	60.0 ± 10.6	*P* < 0.05	63.4 ± 12.6	62.9 ± 11.8	*P* > 0.05
*R*_a_	29.4 ± 8.2	28.8 ± 8.2	30.0 ± 8.1	*P* > 0.05	29.0 ± 8.3	29.8 ± 8.1	*P* > 0.05
*D*_s_	17.4 ± 4.0	19.7 ± 3.0	15.2 ± 3.6	*P* < 0.05	17.3 ± 3.7	17.5 ± 4.3	*P* > 0.05
*D*_a_	12.7 ± 3.3	14.0 ± 3.5	11.3 ± 2.5	*P* < 0.05	12.7 ± 3.5	12.6 ± 3.2	*P* > 0.05
*A*_s_	148.1 ± 5.4	146.7 ± 4.9	149.6 ± 5.6	*P* < 0.05	148.7 ± 5.2	147.6 ± 5.6	*P* > 0.05
*A*_a_	146.6 ± 8.7	145.6 ± 9.4	147.6 ± 7.8	*P* > 0.05	147.5 ± 8.9	145.7 ± 8.4	*P* > 0.05
Curvature in the coronal plane							
Type 2 (convex)							
*H*	4.5 ± 1.7	4.9 ± 1.8	4.0 ± 1.4	*P* > 0.05	4.9 ± 1.6	4.2 ± 1.8	*P* > 0.05
Type 3 (concave)							
*H*	−5.7 ± 1.7	−5.7 ± 1.9	−6.0 ± 0.8	*P* > 0.05	−6.3 ± 1.8	−5.3 ± 1.5	*P* > 0.05
Type 4 (S-shaped)							
*H*_s_	−2.7 ± 1.0	−3.2 ± 1.2	−2.5 ± 0.9	*P* < 0.05	−2.8 ± 1.2	−2.7 ± 0.8	*P* > 0.05
*H*_a_	3.0 ± 1.4	3.2 ± 1.6	2.9 ± 1.3	*P* > 0.05	2.9 ± 1.3	3.1 ± 1.5	*P* > 0.05

**Table 2:** Intramedullary canal (S) and sectional areas (OS) of the clavicle.

Measurements	Total	Male	Female	*P*	Left	Right	*P*
S1	200.3 ± 99.3	220.2 ± 103.5	180.3 ± 91.7	*P* < 0.05	200.2 ± 103.5	200.4 ± 96.0	*P* > 0.05
S2	77.1 ± 32.5	96.4 ± 28.0	57.7 ± 24.4	*P* < 0.05	73.6 ± 31.8	80.6 ± 33.3	*P* > 0.05
S3	53.1 ± 26.0	68.3 ± 25.1	37.8 ± 16.0	*P* < 0.05	53.0 ± 26.2	53.2 ± 26.0	*P* > 0.05
S4	36.1 ± 19.9	46.1 ± 18.6	26.0 ± 15.7	*P* < 0.05	34.2 ± 20.2	37.9 ± 19.5	*P* > 0.05
S5	44.0 ± 23.3	54.8 ± 24.2	33.2 ± 16.5	*P* < 0.05	40.6 ± 21.0	47.4 ± 25.2	*P* > 0.05
S6	65.4 ± 25.8	81.0 ± 22.9	49.8 ± 18.0	*P* < 0.05	63.5 ± 24.1	67.3 ± 27.6	*P* > 0.05
S7	102.8 ± 59.9	131.8 ± 67.5	73.7 ± 31.0	*P* < 0.05	101.9 ± 51.8	103.6 ± 67.5	*P* > 0.05
OS1	326.3 ± 122.7	364.3 ± 114.4	288.2 ± 119.8	*P* < 0.05	330.9 ± 124.6	321.8 ± 121.8	*P* > 0.05
OS2	175.7 ± 51.5	208.0 ± 39.3	143.5 ± 41.2	*P* < 0.05	174.0 ± 53.0	177.4 ± 50.5	*P* > 0.05
OS3	138.0 ± 40.6	167.6 ± 31.3	108.4 ± 23.8	*P* < 0.05	138.7 ± 43.0	137.3 ± 38.4	*P* > 0.05
OS4	121.6 ± 34.7	145.8 ± 27.4	97.3 ± 22.0	*P* < 0.05	120.0 ± 34.8	123.1 ± 34.9	*P* > 0.05
OS5	130.8 ± 40.2	155.3 ± 39.0	106.2 ± 22.6	*P* < 0.05	124.6 ± 38.3	136.9 ± 41.5	*P* > 0.05
OS6	165.3 ± 44.1	197.2 ± 35.4	133.5 ± 24.6	*P* < 0.05	161.4 ± 40.0	169.3 ± 47.9	*P* > 0.05
OS7	210.6 ± 78.9	258.7 ± 75.7	162.5 ± 46.1	*P* < 0.05	207.0 ± 72.0	214.2 ± 84.9	*P* > 0.05

**Table 3:** Comparison of the clavicle morphology with the literature data.

Measurements	Groups	Present study	Mathieu, France	Bernat, Belgium	Bachoura, USA	Daruwalla, Ireland	Huang, USA
*L*	Total	144.2 ± 12.0		149.4 ± 10.3	136.7 ± 10.4		145.0 ± 12.7
	Males	152.9 ± 9.3	152.7 ± 2.9	155.8 ± 7.6		152.33	152.6 ± 10.2
	Females	135.6 ± 7.2	140.2 ± 1.5	142.9 ± 8.4		140.34	137.3 ± 10.2

*T*_s_	Total	20.8 ± 6.0		25.6 ± 3.1			
	Males	21.1 ± 7.5		25.7 ± 3.7			
	Females	20.6 ± 4.1		25.4 ± 3.0			

*T*_m_	Total	10.5 ± 1.9		9.5 ± 1.3			
	Males	11.7 ± 1.9		10.2 ± 1.2		11.69	
	Females	9.3 ± 1.0		8.7 ± 0.9		9.34	

*T*_a_	Total	14.1 ± 3.8		13.6 ± 1.7			
	Males	16.3 ± 3.8		14.6 ± 1.5			
	Females	11.9 ± 2.2		12.7 ± 1.3			

*R*_s_	Total	63.2 ± 12.1			66.4 ± 8.0		
	Males	66.4 ± 12.8	72.1 ± 1.4				
	Females	60.0 ± 10.6	68.7 ± 1.2				

*R*_a_	Total	29.4 ± 8.2			33.5 ± 10.5		
	Males	28.8 ± 8.2	37.1 ± 1.2				
	Females	30.0 ± 8.1	37.8 ± 0.9				

*D*_s_	Total	17.4 ± 4.0					
	Males	19.7 ± 3.0	18.4 ± 1.6				
	Females	15.2 ± 3.6	15.1 ± 1				

*D*_a_	Total	12.7 ± 3.3					
	Males	14.0 ± 3.5	12.3 ± 1.5				
	Females	11.3 ± 2.5	10.2 ± 0.6				
